# Effect of Treating Periodontal Disease in Pregnant Women to Reduce the Risk of Preterm Birth and Low Birth Weight: An Umbrella Review

**DOI:** 10.3390/medicina60060943

**Published:** 2024-06-04

**Authors:** Heber Isac Arbildo-Vega, Tania Padilla-Cáceres, Luz Caballero-Apaza, Fredy Hugo Cruzado-Oliva, Vilma Mamani-Cori, Sheyla Cervantes-Alagón, Hernán Vásquez-Rodrigo, Franz Tito Coronel-Zubiate, Rubén Aguirre-Ipenza, Joan Manuel Meza-Málaga, Sara Antonieta Luján-Valencia, Tania Belú Castillo-Cornock, Katherine Serquen-Olano

**Affiliations:** 1 Department of General Dentistry, Dentistry School, San Martín de Porres University, Chiclayo 14012, Peru; harbildov@usmp.pe (H.I.A.-V.); hvasquezr@usmp.pe (H.V.-R.); tcastilloc@usmp.pe (T.B.C.-C.); kserqueno@usmp.pe (K.S.-O.); 2 Department of Human Medicine, School of Human Medicine, San Martín de Porres University, Chiclayo 14012, Peru; 3 Department of General Dentistry, Dentistry School, University of the Altiplano, Puno 21001, Peru; tpadilla@unap.edu.pe (T.P.-C.); lmcaballero@unap.edu.pe (L.C.-A.); vmamani@unap.edu.pe (V.M.-C.); slcervantes@unap.edu.pe (S.C.-A.); 4 Research Institute in Environmental Sciences, Health and Biodiversity—IICASB, University of the Altiplano, Puno 21001, Peru; 5 Department of Nursing, School of Nursing, University of the Altiplano, Puno 21001, Peru; 6 Department of Stomatology, School of Stomatology, National University of Trujillo, Trujillo 13011, Peru; fcruzado@unitru.edu.pe; 7 Amazonian Andean Research and Development Institute—IIDEAA, University of the Altiplano, Puno 21001, Peru; 8 Department of Dentistry, Dentistry School, Norbert Wiener University, Lima 15046, Peru; 9 Faculty of Health Sciences, Stomatology School, Toribio Rodríguez of Mendoza National University of Amazonas, Chachapoyas 01001, Peru; 10 Faculty of Health Sciences, Continental University, Lima 15046, Peru; raguirrei@continental.edu.pe; 11 Faculty of Dentistry, Dentistry School, Católica de Santa María University, Arequipa 04013, Peru; jmezam@ucsm.edu.pe (J.M.M.-M.); slujan@ucsm.edu.pe (S.A.L.-V.); 12 Faculty of Medicine, Medicine School, Católica de Santa María University, Arequipa 04013, Peru; 13 Postgraduate School, Católica de Santa María University, Arequipa 04013, Peru; 14 Faculty of Health Sciences, Stomatology School, Señor de Sipán University, Chiclayo 14000, Peru

**Keywords:** periodontal disease, preterm birth, low birth weight, pregnancy, pregnant women, periodontitis, review

## Abstract

*Background*: The aim of this review was to evaluate the effects of periodontal disease (PD) treatment in pregnant women to reduce the risk of preterm birth (PB) and low birth weight (LBW) by conducting an umbrella review. *Methods*: A comprehensive search for the literature up to April 2024 was conducted across multiple databases including PubMed, Cochrane Library, Scopus, EMBASE, Scielo, Web of Science, Google Scholar, Proquest Dissertations and Theses, and OpenGrey. We specifically targeted systematic reviews (SRs) with or without meta-analyses, irrespective of language or time constraints, focusing on primary studies examining the effect of PD treatment in pregnant women to reduce the risk of PB and LBW. Various types of non-systematic reviews, intervention studies, observational studies, preclinical and basic research, summaries, comments, case reports, protocols, personal opinions, letters, and posters were excluded from consideration. The quality and overall confidence of the included studies were assessed using the AMSTAR–2 tool. *Results*: After the initial search, 232 articles were identified, of which only 24 met the selection criteria after exclusion. The majority of these studies indicated that periodontal treatment reduces the risk of PB and LBW. *Conclusions*: According to the findings and conclusions drawn from the SRs with a high overall confidence level, PD treatment in pregnant women reduces the risk of PB and LBW.

## 1. Introduction

Periodontal disease (PD) is a destructive chronic inflammatory bacterial infection that affects the supporting tissues of the tooth [1,2], beginning with the accumulation of dental plaque or biofilm with a predominance of Gram-negative anaerobic microorganisms [3] and mediated by the inflammatory response of the host [4].

The initial disease is gingivitis, which produces a localized inflammation of the gums, with redness and/or bleeding. If gingivitis is not treated, it can progress to periodontitis [5]. This progression of bacterial infection leads to the severe destruction of the periodontium and can cause tooth loss [6] and contribute to systemic inflammation [2,7].

Frequent episodes of bacteremia or the dissemination of endotoxins from the periodontal focus could induce the activation of the inflammatory response [8,9] and an intense production of proinflammatory cytokines [10]. Therefore, anti-infective periodontal therapies are considered anti-inflammatory interventions because they reduce exposure to microorganisms and subgingival pathogens [11].

There is evidence that PD is associated with heart disease, diabetes mellitus, chronic obstructive pulmonary disease, rheumatoid arthritis, and adverse pregnancy outcomes [12,13]. Gestational age and birth weight are the most important biological determinants for the possibility of the survival, growth, and development of a newborn [14]. Preterm birth (PB) is defined as the birth of a live baby at less than 37 weeks of gestation [15]; it is one of the main causes of neonatal morbidity and mortality in both developed and developing countries [14]. Between 75% and 80% of perinatal deaths occur in fetuses delivered at less than 37 weeks [16].

The prevalence of PD is high in pregnant mothers (40%) [17]. During pregnancy, due to hormonal factors (high levels of estrogen and progesterone), 50 to 70% of women develop gingivitis, making them more vulnerable to PD than their non-pregnant peers [18]. Although most of these inflammatory changes, such as gingivitis and the localized growth of gingival tissues, disappear within a few months after childbirth, previous epidemiological evidence has suggested that women during pregnancy are more likely to experience periodontal disease caused by a variety of factors [19].

The factors that can induce PB are several; however, the transit of periodontal pathogens, proinflammatory cytokines, and prostaglandins from the periodontal pockets to the fetal–placental unit suggests a plausible hypothesis for the association between PD and pregnancy complications such as PB and LBW [20].

This possibility gave rise to a number of interventional studies, conducted in recent years, to investigate the association between PD and adverse pregnancy outcomes [21], leading to the publication of an umbrella systematic review on this association in 2023 [22], but systematic reviews continue to be published, so an update is necessary.

Therefore, the purpose of this umbrella review was to summarize the available evidence and answer the following specific question: “What do we know so far about the effect of PD treatment on reducing the risk of PB and LBW?” In addition, overall, how reliable are systematic reviews in evaluating this topic?

## 2. Materials and Methods

### 2.1. Protocol and Registration

A protocol was carried out following the Preferred Reporting Items for Systematic Reviews and Meta-Analysis Protocols (PRISMA-P) [23] and registered in the Prospective Registry of Systematic Reviews (PROSPERO) [24]. This record can be publicly accessed using the number CRD42022307617. In addition, the report of this study is based on the Preferred Reporting Items for Overview of Systematic Reviews Checklist (PRIO-harms) [25]. Ethical approval was not required for this umbrella review.

The focused question was formulated using the PICO format (population, intervention, comparison, and outcomes), as detailed below:

- Population: Pregnant women with periodontal disease.
- Intervention: Pregnant women with periodontal disease treatment.
- Comparison: Pregnant women without treatment for periodontal disease.
- Outcomes: Reduction in PB (<37 weeks) and LBW of the newborn (<2500 gm).

### 2.2. Eligibility Criteria and Results of Interest

The studies considered for inclusion were systematic reviews (SRs) with or without meta-analysis and were not limited by time or language. These reviews assessed primary studies that investigated the effect of PD treatment on pregnant women to reduce the risk of PB and LBW. 

Literature or narrative reviews, rapid reviews, intervention studies, observational studies, preclinical and basic research, abstracts, commentaries, case reports, protocols, personal opinions, letters, and posters were excluded.

### 2.3. Sources of Information, Search Strategy, and Additional Search for Primary Studies

On 4 April 2024, an electronic search was conducted across six databases (PubMed, Cochrane Database, Scielo, Web of Science, EMBASE, and Scopus). Grey literature sources were also consulted via Google Scholar, Proquest Dissertations and Theses, and OpenGrey. In addition, the reference lists of the identified studies were scrutinized. Articles retrieved were imported into reference management software (Zotero® 6.0, Center for History and New Media, Fairfax, VA, USA) and any duplicates were eliminated. The search strategy employed for each database is detailed in Table 1.

### 2.4. Data Management and Selection Process

The articles identified were imported into the Rayyan® Online Software (Qatar Research Institute of Computing, Doha, Qatar). The study selection process was conducted in two phases: during phase 1, two reviewers (T.P. and L.C.) independently screened the studies based on their titles and abstracts; subsequently, phase 2 involved the independent full-text assessment by the same two reviewers. In cases of disagreement, a third reviewer (H.A.) was consulted.

### 2.5. Data Collection Process

Information from the studies was collected independently and in duplicate using a table previously devised by two reviewers (F.C.O. and V.C.). The collected data were cross-verified, and any discrepancies were resolved by the third reviewer (H.A.). The extracted information from the selected articles included details such as authors; year of publication; study design; design of the primary studies included; number of studies included in the qualitative and quantitative analysis; results; main conclusions; any conducted meta-analysis; and mentions of what was used or carried out: PRISMA, PROSPERO, and Grading of Recommendations Assessment, Development and Assessment (GRADE).

### 2.6. Assessment of Methodological Quality, Quality of Evidence, and Meta-Bias

The methodological quality of the included SRs was assessed independently and in duplicate by two reviewers (S.C. and H.V.) who were calibrated (Kappa 0.85). This evaluation utilized the AMSTAR-2 checklist (A MeaSurement Tool to Assess Systemic Reviews) [26], which comprises 16 questions with responses categorized as “yes”, “no”, or “partially yes”. The overall confidence rating (high, moderate, low, and critically low) of the studies was assessed following the guidelines proposed by Shea et al. [26].

### 2.7. Summary of Measures

For SRs without meta-analysis, we focused on the results presented as percentages or in general. In SRs that included meta-analysis, we specifically considered outcomes presented as odds ratio (OR) or risk/rate ratio (RR) regarding the effect of PD treatment on reducing the risk of PB and LBW.

### 2.8. Summary of Results

The primary outcomes of the SRs included were condensed, organizing their findings into two categories: reduction in PB and reduction in LBW.

## 3. Results

### 3.1. Review and Selection of Primary Studies

**Figure 1:**
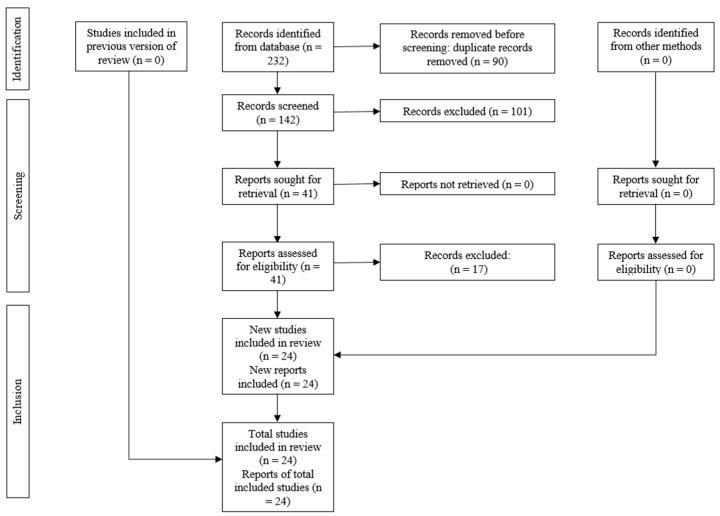
PRISMA flowchart showing the process of inclusion and exclusion of studies.

The electronic search of the database yielded 232 articles, of which 142 remained following the elimination of duplicates. During phase 1, the titles and abstracts of the identified studies were reviewed, resulting in 41 articles deemed suitable for full-text assessment. Finally, 24 SRs were retained for qualitative analysis. The rationales for excluding articles are detailed in Table 2. The complete process of study identification and selection is depicted in Figure 1.

### 3.2. Review and Characteristics of Included Studies

The SRs analyzed in this study spanned from 2003 to 2023 and were conducted in various countries, including Saudi Arabia [43], the United Kingdom [21,44], the United States [45,46,47,48,49], Vietnam [50], Canada [51,52], Australia [53,54], India [55,56], Brazil [10,57,58,59,60], Germany [61], Italy [62], and Greece [63,64]. Additional details regarding the characteristics of these SRs can be found in Table 3.

### 3.3. Assessment of Methodological Quality and Quality of Evidence

Seven SRs [10,21,44,45,46,50,59] were considered to have high confidence, fourteen SRs [43,47,51,52,53,54,55,56,57,58,61,62,63,64] had low confidence, and three SRs [48,49,60] had critically low confidence (Table S1).

### 3.4. Overlap

A total of 274 primary studies were identified across the SRs. Remarkably, approximately 96.75% of these primary studies [20,65,66,67,68,69,70,71,72,73,74,75,76,77,78,79,80,81,82,83,84,85,86,87,88,89], were featured in more than one SR. Specifically, four studies were duplicated; one appeared three times; three appeared four times; two appeared five times; one appeared six times; one appeared seven times; one appeared eight times; one appeared fourteen times; one appeared fifteen times; two appeared sixteen times; one appeared seventeen times; three appeared eighteen times; one appeared nineteen times; two appeared twenty times; and one appeared twenty-one times. Additional details regarding the overlap and characteristics of the primary studies are provided in Table S2.

### 3.5. Synthesis of Results

The summaries of the findings are displayed in Table S3.

#### 3.5.1. Preterm Birth (PB)

Sixteen included SRs [10,21,43,45,46,47,48,49,50,51,53,54,55,56,60,64] reported that PD treatment reduces the risk of PB, while eight SRs [44,52,57,58,59,61,62,63] reported that it did not. Eighteen SRs [10,21,44,45,46,47,50,51,52,53,54,57,58,59,61,62,63,64] meta-analyzed the results and found that the OR ranged from 0.44 (CI: 0.20 to 0.98) [53] to 1.01 (CI: 0.74 to 1.38) [62] and the RR ranged from 0.37 (CI: 0.16 to 0.84) [50] to 0.92 (CI: 0.72 to 1.17) [58]. Alnasser et al. [43], Govindasamy et al. [55], Shah et al. [56], Pimentel Lopes de Oliveira et al. [60], Xiong et al. [48], and Scannapieco et al. [49] reported that PD treatment reduces the risk of PB by up to 50%.

#### 3.5.2. Low Birth Weight (LBW)

Twelve included SRs [43,44,45,46,48,49,50,52,54,55,56,60] reported that PD treatment reduces the risk of LBW, while twelve SRs [10,21,47,51,53,57,58,59,61,62,63,64] reported that it did not. Eighteen SRs [10,21,44,45,46,47,50,51,52,53,54,57,58,59,61,62,63,64] meta-analyzed the results and found that the OR ranged from 0.48 (CI: 0.23 to 1.00) [64] to 1.08 (CI: 0.86 to 1.36) [62] and the RR ranged from 0.44 (CI: 0.31 to 0.65) [52] to 1.03 (CI: 0.76 to 1.40) [58]. Alnasser et al. [43], Govindasamy et al. [55], Shah et al. [56], Pimentel Lopes de Oliveira et al. [60], Xiong et al. [48], and Scannapieco et al. [49] reported that PD treatment reduces the risk of LBW by up to 57%.

## 4. Discussion

Premature birth (PB) and low birth weight (LBW) are public health problems of great importance worldwide today. According to reports from the World Health Organization (WHO) and the United Nations Children’s Fund (UNICEF), there have been no significant changes in the incidence of PBs between 2010 (13.4 million) and 2020 (13.8 million) [90]. Similarly, the rate of LBW babies has also not shown variations over the last decade, with this stagnation being attributed mainly to the lack of comprehensive prenatal care for pregnant women [91]. WHO recommends expanding prenatal care to improve the health of both mothers and newborns, through five key interventions: nutritional interventions; physical health checks; maternal and fetal assessments; preventive measures; and health system interventions, including oral health care [92]. Periodontal disease in pregnancy should be considered an important risk factor for premature births; its control should be before and during pregnancy as long as it is preventable and treatable to reduce premature neonates [43].

In 1996, Offenbacher was the first to report the association between PD in pregnant women and an increased risk of PB and LBW in babies [93], with this connection being confirmed in several review studies [27,28,30]. This is because hormonal changes during pregnancy, such as elevated levels of estrogen and progesterone, increase vascular permeability in the gums, which facilitates the spread of bacteria and their products, especially anaerobic Gram-negative bacteria, to the fetus [94]. Some bacteria responsible for periodontal disease cross the placenta and reach the fetus, interfering with its growth, and due to the increase in prostaglandins, they increase the systemic inflammatory state of the mother, slowing down the growth of the fetus [95]. Therefore, it is important for doctors to advise pregnant women who present symptoms of PD to visit the dentist for treatment [43], to cure or intercept periodontal pathology, also considering care from the preventive phase [51].

In recent years, there has been increased interest among oral health researchers in exploring the possible relationship between PD treatment and adverse outcomes, such as PB and LBW, in pregnant women. It has been observed that non-surgical periodontal treatment during pregnancy can effectively reduce periodontal inflammation and local cytokine levels [84]. In addition, the use of antimicrobial mouthwashes during pregnancy, such as chlorhexidine or cetylpyridinium chloride, called conventional treatments for periodontal disease, has a protective association of preventing premature birth; it can also reduce bacterial plaque, gingival bleeding, and inflammation gingival [45]. This could have a positive impact on the main proposed pathways of inflammatory migration towards the fetal–placental unit. These routes include the direct route, where oral microorganisms and/or their components reach the fetal–placental unit through blood circulation from the oral cavity, and the indirect route, where inflammatory mediators produced in the periodontal tissues circulate to the liver and increase the systemic inflammatory state, reflecting in acute phase protein responses, such as C-reactive protein, which could then affect the fetal–placenta unit [49,50,51,53]. So, periodontal treatment during pregnancy is related to a decrease in the levels of periodontal inflammatory biomarkers and some blood serum, but it is not clear if this non-surgical treatment influences premature birth [10,44].

An umbrella review conducted in 2018 that covered 18 SRs on the effect of periodontal treatment in pregnant women and adverse obstetric outcomes highlighted that non-surgical periodontal treatment contributed to reducing the incidence of adverse outcomes such as LBW, PB, and pre-eclampsia [96]. Furthermore, Chen et al. [97] also found a positive association between periodontal treatment and the risk of adverse birth outcomes. These results were confirmed in a longitudinal cohort study conducted in Chile, which included 870 people, where the treatment of PD significantly reduced the rate of PB and LBW in women with pregnancy-associated gingivitis [74]. However, in a recent analysis, Khan et al. [22] evaluated 17 SRs and concluded that the evidence on this topic is inconclusive, because there are insufficient clinical trials, causing them to present high possibilities of bias in the research, because it is not possible to evaluate some specific aspects such as the diagnosis, extension, and severity of the disease [59].

In contrast to Machado et al. [98], who analyzed the systematic reviews and only described the main findings on the association between maternal periodontitis and adverse pregnancy outcomes (APOs), this review performed an analysis of the meta-analytic estimates from all systematic reviews with meta-analyses from inception to February 2023. These results allow for the generation of solid scientific evidence maps that will contribute decisively to developing oral and periodontal care strategies for pregnant women, with the primary goal of minimizing pregnancy complications. Additionally, unlike the previous review, this review did evaluate the methodological quality of the primary studies included, thus avoiding an important methodological limitation.

The present study carried out a comprehensive search of the literature in order to compile and analyze the available SRs on the relationship between the treatment of PD and cases of PB and LBW, identifying 24 SRs that met the established selection criteria. Although SRs represent a solid source of scientific evidence, it is crucial to be cautious when interpreting their results due to the possibility of bias. The SRs included in this study presented certain limitations related to the selected primary studies, which focused on different types of study and different definition criteria for PD (gingivitis or periodontitis).

Some of the studies analyzed in this study exhibited a high level of confidence, which could reinforce the validity of the results and conclusions obtained. However, the continued presence of SRs with lower confidence levels highlights the urgent need to apply greater rigor in the execution of research related to this topic.

The assessment of the methodological quality of the included SRs was carried out using the AMSTAR-2 tool, which is recognized and widely used today. Deficiencies in critical domains 2 and 7 of this tool were identified in some studies. These deficiencies include the lack of description of the methodology used to prepare the review before its execution, as well as the absence of a list of excluded studies with their corresponding justification. These findings highlight the importance of addressing these aspects in future SRs. Furthermore, it is necessary to be cautious when interpreting the results of the SRs, since approximately 96.75% of the included primary studies overlap in multiple reviews, which could lead to repetitions in the evaluation of the same data. This situation could distort the perception of the amount of research carried out in this area. Although, it would be beneficial to conduct new SRs to address these methodological limitations and the high degree of overlap between existing reviews [99].

### 4.1. Evidence Summary

In this review, we seek to clarify the relationship between the treatment of PD and PB/LBW by analyzing SRs and meta-analyses available on the topic. The SRs examined in this study suggest a positive and direct association between the treatment of PD and the reduction in the risk of PB/LBW, supporting previous findings by other authors such as Rangel-Rincón et al. [96] and López et al. [74]. However, researchers such as Machado et al. [98] and Lavigne and Forrest [100] have raised doubts about this reduction and point to the need for future research to clarify this association.

Furthermore, it is highlighted that the use of mouthwashes such as chlorhexidine, in combination with conventional treatments for PE during pregnancy, is protectively associated with the prevention of adverse birth outcomes [10,45].

### 4.2. Implications for Clinical Practice

Oral health professionals have a responsibility to educate patients about how the non-surgical treatment of PD and the use of mouthwashes can reduce the risk of adverse outcomes in pregnant women. In the context of personalized medicine, it would be prudent to incorporate preventive dental consultations to detect PD early and treat it before inflammatory migration to the fetal–placental unit occurs. Close collaboration with neonatologists, obstetrician–gynecologists, and other specialists is suggested for the optimal management of pregnant patients and PB/LBW infants. In addition, university educational institutions should strengthen the curricula of dental schools, including a comprehensive perspective on health in pregnant women.

### 4.3. Implications for Research

This review underscores the imperative to enhance the quality of SRs within this domain. Authors are advised to employ quality assessment tools to steer the formulation of future SRs, thereby ensuring methodological robustness. Additionally, there is a strong emphasis on conducting primary studies with elevated methodological rigor to yield more dependable outcomes. In terms of future research endeavors in this realm, it is suggested to establish standardized diagnostic criteria for PD, undertake high-caliber prospective studies with expanded sample sizes and consistent metrics, and delve deeper into investigations to elucidate the precise mechanisms and extent of the association between PD and adverse obstetric outcomes.

## 5. Conclusions

According to the findings and conclusions drawn from the SR with a high overall confidence level, PD treatment in pregnant women reduces the risk of PB and LBW.

## Figures and Tables

**Table 1:** Search strategies for each database.

Database	Search Strategy	Number of Studies
PubMed	((“periodontal disease treatment“) OR (periodontitis) OR (gingivitis)) AND ((“preterm birth“) OR (“low birth weight“) OR (“perinatal outcomes“) OR (“premature labor“) OR (“adverse pregnancy outcomes“)) AND ((“systematic review“) OR (“meta-analysis“))	77
Cochrane Database	#1 MeSH descriptor: [Periodontal Diseases] explode all trees #2 MeSH descriptor: [Periodontitis] explode all trees #3 MeSH descriptor: [Gingivitis] explode all trees #4 (periodontal disease treatment) OR (periodontitis) OR (gin-givitis) (Word variations have been searched) #5 #1 OR #2 OR #3 OR #4 #6 MeSH descriptor: [Premature Birth] explode all trees #7 MeSH descriptor: [Infant, Low Birth Weight] explode all trees #8 MeSH descriptor: [Obstetric Labor, Premature] explode all trees #9 (preterm birth) OR (low birth weight) OR (perinatal out-comes) OR (premature labor) OR (adverse pregnancy out-comes) (Word variations have been searched) #10 #6 OR #7 OR #8 OR #9 #11 #5 AND #10	9
Scielo	((((“periodontal disease“) OR (treatment) (periodontitis) OR (gingivitis))) AND (((“preterm birth“) OR (“low birth weight“) OR (“perinatal outcomes“) OR (“premature labor“) OR (“ad-verse pregnancy outcomes“)))) AND ((“systematic review“))	11
Scopus	TITLE-ABS-KEY (((“periodontal disease treatment“) OR periodontitis) OR gingivitis) AND TITLE-ABS-KEY (((((“preterm birth“) OR “low birth weight“) OR “perinatal outcomes“) OR “premature labor“) OR “adverse pregnancy outcomes“) AND TITLE-ABS-KEY (“systematic review“) AND (LIMIT-TO (SUBJAREA, “DENT“)) AND (LIMIT-TO (DOCTYPE, “re“))	15
Web of Science	((TS = (((“periodontal disease treatment“) OR (periodontitis) OR (gingivitis)))) AND TS = (((“preterm birth“) OR (“low birth weight“) OR (“perinatal outcomes“) OR (“premature labor“) OR (“adverse pregnancy outcomes“)))) AND TS = ((“systematic re-view“))	42
EMBASE	(‘periodontal disease treatment’:ti,ab,kw OR ‘periodontitis’:ti,ab,kw OR ‘gingivitis’:ti,ab,kw) AND (‘preterm birth’:ti,ab,kw OR ‘low birth weight’:ti,ab,kw OR ‘perinatal outcomes’:ti,ab,kw OR ‘premature labor’:ti,ab,kw OR ‘adverse pregnancy outcomes’:ti,ab,kw) AND (‘systematic review’:ti,ab,kw)	36
Google Scholar	allintitle: “periodontal disease treatment“ “preterm birth“ “sys-tematic review“ allintitle: “periodontitis“ “preterm birth“ “systematic review“	8
Proquest Dissertations and Theses	(“periodontal disease treatment“ OR “periodontitis“ OR “gingivitis“) AND (“preterm birth“ OR “low birth weight“ OR “perinatal outcomes“ OR “premature labor“ OR “adverse pregnancy outcomes“) AND (“systematic review“ OR “meta-analysis“) NOT (“umbrella“ OR “overview“ OR “obesity“ OR “relationship“ OR “in vivo“ OR “dementia“ OR “knowledge“ OR “narrative“ OR “in vitro“)	34
OpenGrey	((“periodontal disease treatment“) OR (periodontitis) OR (gingivitis)) AND ((“preterm birth“) OR (“low birth weight“) OR (“perinatal outcomes“) OR (“premature labor“) OR (“adverse pregnancy outcomes“)) AND ((“systematic review“) OR (“meta-analysis“))	0

**Table 2:** Reason for exclusion of included studies.

Authors	Reason for Exclusion
Karimi et al. [27], Padilla-Cáceres et al. [28], Oralkhan et al. [29], Zhang et al. [30], Porto et al. [31], Manrique-Corredor et al. [32], Teshome et al. [4], Corbella et al. [33], Ide et al. [34], Shanthi et al. [35], Corbella et al. [36], Konopka et al. [37], Chambrone et al. [38], Vergnes et al. [39], Vettore et al. [40], Khader et al. [41]	Non-treatment of PD
Oates et al. [42]	Data reported in log(OR)

**Table 3:** Characteristics of included studies.

Authors	Year	Study Design	Country	Included Study Design	Number of Studies in the Qualitative Analysis	Number of Studies in the Quantitative Analysis	Outcomes		Conclusions
Alnasser et al. [43]	2023	SR	Saudi Arabia	RCT	20	0	PB and LBW	11 studies showed a positive impact of periodontitis therapy in reducing the risk of adverse pregnancy outcomes	Periodontal therapy has a reducing effect on adverse pregnancy outcomes in pregnant women with periodontitis.
Orlandi et al. [21]	2022	SR and MA	United Kingdom	RCT	16	16	PB	RR = 0.77 (0.6–0.98)	Treatment of periodontitis results in improvements in systemic health including reduction in PBs and LBW of the newborn.
							LBW	RR = 0.77 (0.57–1.02)	
Merchant et al. [45]	2022	SR and MA	United States	RCT	12	12	PB	RR = 0.56 (0.34–0.93)	Adding chlorhexidine to conventional treatment of maternal periodontitis has a protective association in preventing adverse birth outcomes.
							LBW	RR = 0.47 (0.32–0.68)	
Le et al. [50]	2022	SR and MA	Vietnam	RCT	20	18	PB	RR = 0.37 (0.16–0.84)	Incorporating mouthwash alongside scaling and root planning during pregnancy as part of PD treatment significantly enhances perinatal outcomes.
							LBW	RR = 0.54 (0.40–0.74)	
Bi et al. [51]	2021	SR and MA	Canada	RCT	20	19	PB	RR = 0.78 (0.62–0.98)	Periodontal treatment during pregnancy reduces risks of perinatal mortality and PB and improves birth weight.
							LBW	RR = 0.76 (0.56–1.03)	
Le et al. [53]	2021	SR an MA	Australia	RCT and CT	3	3	PB	OR = 0.44 (0.20–0.98)	The results of the meta-analysis indicate that the treatment of gingivitis in pregnant women increases the birth weight of the newborn and reduces PB.
							LBW	OR = 0.92 (0.38–2.21)	
Govindasamy et al. [55]	2020	SR	India	RCT	19	0	PB and LBW	Twelve studies showed the positive influence of periodontal therapy on pregnancy outcomes.	Non-surgical treatment for PD is considered safe during pregnancy. While it may not entirely eliminate adverse pregnancy outcomes, it could be advised as a component of prenatal health.
Iheozor—Ejiofor et al. [44]	2017	SR and MA	United Kingdom	RCT	15	15	PB	RR = 0.87 (0.70–1.10)	The effect of periodontal treatment during pregnancy on PB remains uncertain, but it might lower the incidence of LBW.
							LBW	RR = 0.67 (0.48–0.95)	
da Silva et al. [10]	2017	SR and MA	Brazil	RCT	4	4	PB	RR = 0.54 (0.38–0.77)	Non-surgical periodontal therapy during pregnancy reduced PB but not LBW.
							LBW	RR = 0.78 (0.5–1.21)	
Schwendicke et al. [61]	2015	SR and MA	Germany	RCT	13	13	PB	OR = 0.79 (0.57–1.10)	Providing periodontal treatment to pregnant women could potentially reduce the risks of perinatal outcomes, especially in high-risk mothers.
							LBW	OR = 0.69 (0.43–1.13)	
Shah et al. [56]	2013	SR	India	RCT	13	0	PB and LBW	Two studies found significant differences in the incidence of LBW and four studies for PB.	Treatment of periodontitis during pregnancy improves pregnancy outcomes in terms of PB and LBW.
Boutin et al. [52]	2013	SR and MA	Canada	RCT	12	12	PB	RR = 0.89 (0.73–1.08)	Periodontal treatment as a standalone intervention does not demonstrate efficacy in decreasing the PB rate among women with PD.
							LBW	RR = 0.44 (0.31–0.65)	
Kim et al. [46]	2012	SR and MA	United States	RCT	12	12	PB	RR = 0.66 (0.54–0.80)	Periodontal treatment reduces the risk of PB and LBW in pregnant women with periodontitis only for high-risk groups.
							LBW	RR = 0.48 (0.30–0.78)	
Rosa et al. [57]	2012	SR and MA	Brazil	RCT	13	13	PB	RR = 0.9 (0.68–1.19)	Primary periodontal care during pregnancy cannot reduce the rate of PB or LBW.
							LBW	RR = 0.92 (0.71–1.20)	
Corbella et al. [62]	2012	SR and MA	Italy	RCT	11	5	PB	OR = 1.01 (0.74–1.38)	Non-surgical periodontal therapy is considered safe for pregnant women; however, our findings did not reveal evidence supporting its effectiveness in reducing the occurrence of PB or LBW.
							LBW	OR = 1.08 (0.86–1.36)	
George et al. [54]	2011	SR and MA	Australia	RCT	10	10	PB	OR = 0.65 (0.45–0.93)	The collective evidence indicates that treating PD during pregnancy might lower the occurrence of PB and LBW.
							LBW	OR = 0.53 (0.31–0.92)	
Fogacci et al. [58]	2011	SR and MA	Brazil	RCT	10	10	PB	RR = 0.92 (0.72–1.17)	The findings of this meta-analysis contradict the idea that periodontal therapy decreases the rate of PB and LBW.
							LBW	RR = 1.03 (0.76–1.40)	
Chambrone et al. [59]	2011	SR and MA	Brazil	RCT	13	11	PB	RR = 0.88 (0.72–1.09)	Periodontal treatment showed no evidence of decreasing the likelihood of PB and/or LBW.
							LBW	RR = 0.78 (0.53–1.17)	
Polyzos et al. [63]	2010	SR and MA	Greece	RCT	11	11	PB	OR = 0.93 (0.79–1.10)	It is improbable that periodontal treatment during pregnancy lowers the risk of PB or LBW.
							LBW	OR = 0.85 (0.70–1.04)	
Uppal et al. [47]	2010	SR and MA	United States	RCT	10	10	PB	OR = 0.59 (0.39–0.88)	The results do not support the hypothesis of a reduction in PB or LBW in women who are treated for periodontal disease during pregnancy.
							LBW	OR = 0.72 (0.44–1.17)	
Pimentel Lopes de Oliveira et al. [60]	2010	SR	Brazil	RCT	7	0	PB and LBW	Reductions of PB ranged from 0.8% to 28.01%, while reduction of LBW ranged from 0.44% to 33%.	The non-surgical periodontal treatment in pregnant women reduces the incidence of PB with LBW.
Polyzos et al. [64]	2009	SR and MA	Greece	RCT	7	7	PB	OR = 0.55 (0.35–0.86)	Treatment involving scaling and/or root planning during pregnancy substantially decreases the incidence of PB and may potentially lower the occurrence of infants with LBW.
							LBW	OR = 0.48 (0.23–1.00)	
Xiong et al. [48]	2006	SR	United States	RCT and CT	3	0	PB and LBW	Three clinical trial studies indicate that oral prophylaxis and periodontal treatment may result in a 57% decrease in LBW cases and a 50% decrease in PB occurrences.	Treatment of periodontal disease reduces the risk of LBW and PB.
Scannapieco et al. [49]	2003	SR	United States	RCT and CT	3	0	PB and LBW	Periodontal treatment reduces PB and LBW	Periodontal disease and preterm birth: results of a pilot intervention study

SR = systematic review; MA = meta-analysis; CT = clinical trial; RCT = randomized clinical trial; PD = periodontal disease; PB = preterm birth; LBW = low birth weight; OR = odds ratio; RR = risk/rate ratio.

## Data Availability

No new data were created or analyzed in this study.

## Supplementary Materials

The following supporting information can be downloaded at: https://www.mdpi.com/article/10.3390/medicina60060943/s1, Table S1: Risk of bias analysis of included studies; Table S2: Overlapping of primary studies in systematic reviews; Table S3: Synthesis of the results of the included studies.
