# Supplementary material for: Effect of Treating Periodontal Disease in Pregnant Women to Reduce the Risk of Preterm Birth and Low Birth Weight: An Umbrella Review
Source: Medicina (Kaunas). 2024 Jun 4;60(6):943. doi: 10.3390/medicina60060943 (PMC11205593; doi:10.3390/medicina60060943)
Supplement: Supplementary file 1 [file medicina-60-00943-s001.zip › medicina-3022264-supplementary.pdf]

Table S1. Risk of bias analysis of included studies

Authors	Year	AMSTAR-2																Overall Confidence
		1	2 *	3	4 *	5	6	7 *	8	9 *	10	11 *	12	13 *	14	15 *	16	
Alnasser et al. [1]	2023	Yes	No	Yes	Yes partial	Yes	Yes	Yes	Yes	Yes	Yes	No meta-analysis		Yes	Yes	No meta-analysis	Yes	Low
Orlandi et al. [2]	2022	Yes	Yes	Yes	Yes partial	Yes	Yes	Yes	Yes	Yes	Yes	Yes	Yes	Yes	Yes	Yes	Yes	High
Merchant et al. [3]	2022	Yes	Yes	Yes	Yes partial	Yes	Yes	Yes partial	Yes	Yes	Yes	Yes	Yes	Yes	Yes	Yes	Yes	High
Le et al. [4]	2022	Yes	Yes partial	Yes	Yes partial	Yes	Yes	Yes	Yes	Yes	Yes	Yes	Yes	Yes	Yes	Yes	Yes	High
Bi et al. [5]	2021	Yes	No	Yes	Yes partial	Yes	Yes	Yes partial	Yes	Yes	Yes	Yes	Yes	Yes	Yes	Yes	Yes	Low
Le et al. [6]	2021	Yes	No	Yes	Yes partial	Yes	Yes	Yes partial	Yes	Yes	Yes	Yes	Yes	Yes	Yes	Yes	Yes	Low
Govindasamy et al. [7]	2020	No	No	Yes	Yes partial	Yes	Yes	Yes	Yes	Yes	Yes	No meta-analysis		Yes	Yes	No meta-analysis	Yes	Low
Iheozor-Ejiofor et al. [8]	2017	Yes	Yes	Yes	Yes partial	Yes	Yes	Yes	Yes	Yes	Yes	Yes	Yes	Yes	Yes	Yes	Yes	High
da Silva et al. [9]	2017	Yes	Yes	Yes	Yes	Yes	Yes	Yes	Yes	Yes	Yes	Yes	Yes	Yes	Yes	Yes	Yes	High
Schwendicke et al. [10]	2015	No	Yes partial	Yes	Yes partial	Yes	Yes	No	Yes	Yes	Yes	Yes	Yes	Yes	Yes	Yes	Yes	Low
Shah et al. [11]	2013	No	No	Yes	Yes partial	Yes	Yes	Yes	Yes	Yes	Yes	No meta-analysis		Yes	Yes	No meta-analysis	Yes	Low
Boutin et al. [12]	2013	Yes	No	Yes	Yes partial	Yes	Yes	Yes	Yes	Yes	Yes	Yes	Yes	Yes	Yes	Yes	Yes	Low
Kim et al. [13]	2012	Yes	Yes	Yes	Yes partial	Yes	Yes	Yes	Yes	Yes	Yes	Yes	Yes	Yes	Yes	Yes	Yes	High
Rosa et al. [14]	2012	Yes	No	Yes	Yes partial	Yes	Yes	Yes	Yes	Yes partial	Yes	Yes	Yes	Yes	Yes	Yes	Yes	Low
Corbella et al. [15]	2012	No	No	Yes	Yes partial	Yes	Yes	Yes partial	Yes	Yes partial	Yes	Yes	Yes	Yes	Yes	Yes	Yes	Low
George et al. [16]	2011	Yes	No	Yes	Yes	Yes	Yes	Yes	Yes	Yes partial	Yes	Yes	Yes	Yes	Yes	Yes	Yes	Low
Fogacci et al. [17]	2011	Yes	No	Yes	Yes partial	Yes	Yes	Yes	Yes	Yes	Yes	Yes	Yes	Yes	Yes	Yes	Yes	Low
Chambrone et al. [18]	2011	Yes	Yes partial	Yes	Yes partial	Yes	Yes	Yes	Yes	Yes	Yes	Yes	Yes	Yes	Yes	Yes	Yes	High
Polyzos et al. [19]	2010	No	No	Yes	Yes partial	Yes	Yes	Yes	Yes	Yes	Yes	Yes	Yes	Yes	Yes	Yes	Yes	Low
Uppal et al. [20]	2010	No	No	Yes	Yes partial	Yes	Yes	Yes partial	Yes	Yes	Yes	Yes	Yes	Yes	Yes	Yes	Yes	Low
Pimentel Lopes de Oliveira et al. [21]	2010	No	No	Yes	No	Yes	Yes	No	Yes	Yes partial	Yes	No meta-analysis		Yes	Yes	No meta-analysis	Yes	Critically low
Polyzos et al. [22]	2009	No	No	Yes	Yes partial	Yes	Yes	Yes	Yes	Yes partial	Yes	Yes	Yes	Yes	Yes	Yes	Yes	Low
Xiong et al. [23]	2006	No	No	Yes	Yes partial	No	No	No	Yes	No	Yes	No meta-analysis		Yes	Yes	No meta-analysis	Yes	Critically low
Scannapieco et al. [24]	2003	Yes	No	Yes	Yes partial	Yes	Yes	No	Yes	Yes partial	Yes	No meta-analysis		Yes	Yes	No meta-analysis	Yes	Critically low

AMSTAR = A Measurement Tool to Assess Systematic Reviews. Items AMSTAR-2: 1. Do the research questions and inclusion criteria for the review include the PICO components ?; 2. Does the review report contain an explicit statement that the review methods were established prior to its

performance and does it justify any significant deviation from the protocol ?; 3. Did the review authors explain their decision about the study designs to include in the review ?; 4. Did the review authors use a comprehensive literature search strategy ?; 5. Did the review authors perform the study selection in duplicate ?; 6. Did the review authors perform data extraction in duplicate ?; 7. Did the review authors provide a list of excluded studies and justify the exclusions ?; 8. Did the review authors describe the included studies in sufficient detail ?; 9. Did the review authors use a satisfactory technique to assess the risk of bias of the individual studies included in the review ?; 10. Did the review authors report the sources of funding for the studies included in the review ?; 11. If a meta-analysis was performed, did the review authors use appropriate methods for the statistical combination of results ?; 12. If a meta-analysis was performed, did the review authors assess the potential impact of risk of bias in individual studies on the results of the meta-analysis or other evidence synthesis ?; 13. Did the review authors consider the risk of bias of individual studies when interpreting / discussing the results of the review ?; 14. Did the review authors provide a satisfactory explanation and discuss any observed heterogeneity in the review results ?; 15. If quantitative synthesis was performed, did the review authors carry out an adequate investigation of publication bias (small study bias) and discuss its likely impact on the results of the review ?; 16. Did the review authors report any potential sources of conflict of interest, including any funding received to carry out the review? * = critical domain

Table S2. Overlapping of primary studies in systematic reviews

Primary Studies	Systematic Reviews That Included the Primary Studies	Times That Primary Studies Were Included
López et al. [25]	Alnasser et al. [1], Orlandi et al. [2], Merchant et al. [3], Le et al. [4], Bi et al. [5], Govindasamy et al. [7], Iheozor-Ejiofor et al. [8], Schwendicke et al. [10], Shah et al. [11], Boutin et al. [12], Kim et al. [13], Rosa et al. [14], Corbella et al. [15], George et al. [16], Fogacci et al. [17], Chambrone et al. [18], Polyzos et al. [19], Uppal et al. [20], Polyzos et al. [22], Xiong et al. [23], Scannapieco et al. [24]	21
Tarannum et al. [26]	Alnasser et al. [1], Orlandi et al. [2], Merchant et al. [3], Le et al. [4], Bi et al. [5], Govindasamy et al. [7], Iheozor-Ejiofor et al. [8], Schwendicke et al. [10], Shah et al. [11], Boutin et al. [12], Kim et al. [13], Rosa et al. [14], Corbella et al. [15], George et al. [16], Fogacci et al. [17], Chambrone et al. [18], Polyzos et al. [19], Uppal et al. [20], Pimentel Lopes de Oliveira et al. [21], Polyzos et al. [22]	20
Michalowicz et al. [27]	Alnasser et al. [1], Orlandi et al. [2], Merchant et al. [3], Le et al. [4], Bi et al. [5], Govindasamy et al. [7], Iheozor-Ejiofor et al. [8], Schwendicke et al. [10], Shah et al. [11], Boutin et al. [12], Kim et al. [13], Rosa et al. [14], Corbella et al. [15], George et al. [16], Fogacci et al. [17], Chambrone et al. [18], Polyzos et al. [19], Uppal et al. [20], Pimentel Lopes de Oliveira et al. [21], Polyzos et al. [22]	20
Sadatmansouri et al. [28]	Alnasser et al. [1], Orlandi et al. [2], Merchant et al. [3], Le et al. [4], Bi et al. [5], Govindasamy et al. [7], Iheozor-Ejiofor et al. [8], Schwendicke et al. [10], Shah et al. [11], Boutin et al. [12], Kim et al. [13], Rosa et al. [14], Corbella et al. [15], George et al. [16], Fogacci et al. [17], Chambrone et al. [18], Polyzos et al. [19], Pimentel Lopes de Oliveira et al. [21], Polyzos et al. [22]	19
Newnham et al. [29]	Alnasser et al. [1], Orlandi et al. [2], Merchant et al. [3], Le et al. [4], Bi et al. [5], Govindasamy et al. [7], Iheozor-Ejiofor et al. [8], Schwendicke et al. [10], Shah et al. [11], Boutin et al. [12], Kim et al. [13], Rosa et al. [14], Corbella et al. [15], George et al. [16], Fogacci et al. [17], Chambrone et al. [18], Polyzos et al. [19], Uppal et al. [20]	18
Offenbacher et al. [30]	Orlandi et al. [2], Le et al. [4], Bi et al. [5], Govindasamy et al. [7], Iheozor-Ejiofor et al. [8], da Silva et al. [9], Schwendicke et al. [10], Boutin et al. [12], Kim et al. [13], Rosa et al. [14], Corbella et al. [15], George et al. [16], Fogacci et al. [17], Chambrone et al. [18], Polyzos et al. [19], Uppal et al. [20], Pimentel Lopes de Oliveira et al. [21], Polyzos et al. [22]	18
Offenbacher et al. [31]	Alnasser et al. [1], Orlandi et al. [2], Merchant et al. [3], Le et al. [4], Bi et al. [5], Govindasamy et al. [7], Iheozor-Ejiofor et al. [8], Schwendicke et al. [10], Shah et al. [11], Boutin et al. [12], Kim et al. [13], Rosa et al. [14], Corbella et al. [15], George et al. [16], Fogacci et al. [17], Chambrone et al. [18], Polyzos et al. [19], Uppal et al. [20]	18
Jeffcoat et al. [32]	Orlandi et al. [2], Bi et al. [5], Govindasamy et al. [7], Iheozor-Ejiofor et al. [8], Schwendicke et al. [10], Shah et al. [11], Boutin et al. [12], Kim et al. [13], Rosa et al. [14], Corbella et al. [15], George et al. [16], Fogacci et al. [17], Chambrone et al. [18], Polyzos et al. [19], Uppal et al. [20], Polyzos et al. [22], Xiong et al. [23]	17
Radnai et al. [33]	Alnasser et al. [1], Orlandi et al. [2], Merchant et al. [3], Le et al. [4], Bi et al. [5], Govindasamy et al. [7], Iheozor-Ejiofor et al. [8], Schwendicke et al. [10], Shah et al. [11], Kim et al. [13], Corbella et al. [15], George et al. [16], Fogacci et al. [17], Chambrone et al. [18], Uppal et al. [20], Pimentel Lopes de Oliveira et al. [21]	16
López et al. [34]	Alnasser et al. [1], Merchant et al. [3], Bi et al. [5], Le et al. [6], Govindasamy et al. [7], Iheozor-Ejiofor et al. [8], Shah et al. [11], Boutin et al. [12], Rosa et al. [14], Corbella et al. [15], George et al. [16], Chambrone et al. [18], Polyzos et al. [19], Uppal et al. [20], Pimentel Lopes de Oliveira et al. [21], Polyzos et al. [22]	16
Macones et al. [35]	Alnasser et al. [1], Orlandi et al. [2], Le et al. [4], Bi et al. [5], Govindasamy et al. [7], Iheozor-Ejiofor et al. [8], Schwendicke et al. [10], Shah et al. [11], Boutin et al. [12],	15

	Kim et al. [13], Rosa et al. [14], Fogacci et al. [17], Chambrone et al. [18], Polyzos et al. [19], Uppal et al. [20]	
Oliveira et al. [36]	Alnasser et al. [1], Orlandi et al. [2], Merchant et al. [3], Le et al. [4], Bi et al. [5], Govindasamy et al. [7], Iheozor-Ejiofor et al. [8], Schwendicke et al. [10], Shah et al. [11], Boutin et al. [12], Kim et al. [13], Rosa et al. [14], Chambrone et al. [18], Polyzos et al. [19]	14
Pirie et al. [37]	Alnasser et al. [1], Orlandi et al. [2], Le et al. [4], Bi et al. [5], Govindasamy et al. [7], Iheozor-Ejiofor et al. [8], da Silva et al. [9], Schwendicke et al. [10]	8
Weidlich et al. [38]	Alnasser et al. [1], Bi et al. [5], Govindasamy et al. [7], Schwendicke et al. [10], Shah et al. [11], Boutin et al. [12], Rosa et al. [14]	7
Jeffcoat et al. [39]	Alnasser et al. [1], Orlandi et al. [2], Le et al. [4], Bi et al. [5], Kim et al. [13], Chambrone et al. [18]	6
Gazolla et al. [40]	Le et al. [4], Govindasamy et al. [7], Shah et al. [11], Corbella et al. [15], Pimentel Lopes de Oliveira et al. [21]	5
Khairnar et al. [41]	Orlandi et al. [2], Le et al. [4], Bi et al. [5], Govindasamy et al. [7], da Silva et al. [9]	5
Herrera et al. [42]	Alnasser et al. [1], Merchant et al. [3], Bi et al. [5], Iheozor-Ejiofor et al. [8]	4
Sant'Ana et al. [43]	Alnasser et al. [1], Bi et al. [5], Govindasamy et al. [7], Rosa et al. [14]	4
Caneiro-Queija et al. [44]	Alnasser et al. [1], Orlandi et al. [2], Merchant et al. [3], Le et al. [4]	4
Reddy et al. [45]	Orlandi et al. [2], Le et al. [4], Govindasamy et al. [7]	3
Mitchell-Lewis et al. [46]	Xiong et al. [23], Scannapieco et al. [24]	2
Farrel et al. [47]	Bi et al. [5], Iheozor-Ejiofor et al. [8]	2
Novák et al. [48]	Le et al. [4], Bi et al. [5]	2
Penova-Vaselinovic et al. [49]	Govindasamy et al. [7], da Silva et al. [9]	2
Novák et al. [50]	Le et al. [4], Le et al. [6]	2

Table S3. Synthesis of the results of the included studies

**Authors**		**Outcome**	**Association**
Alnasser et al. [1]	PB and LBW	11 studies showed a positive impact of periodontitis therapy in reducing the risk of adverse pregnancy outcomes	Yes
Orlandi et al. [2]	PB	RR = 0.77 (0.6 – 0.98)	Yes
	LBW	RR = 0.77 (0.57 – 1.02)	No
Merchant et al. [3]	PB	RR = 0.56 (0.34 – 0.93)	Yes
	LBW	RR = 0.47 (0.32 – 0.68)	Yes
Le et al. [4]	PB	RR = 0.37 (0.16 – 0.84)	Yes
	LBW	RR = 0.54 (0.40 – 0.74)	Yes
Bi et al. [5]	PB	RR = 0.78 (0.62 – 0.98)	Yes
	LBW	RR = 0.76 (0.56 – 1.03)	No
Le et al. [6]	PB	OR = 0.44 (0.20 – 0.98)	Yes
	LBW	OR = 0.92 (0.38 – 2.21)	No
Govindasamy et al. [7]	PB and LBW	Twelve studies showed the positive influence of periodontal therapy on pregnancy outcomes	Yes
Iheozor – Ejiofor et al. [8]	PB	RR = 0.87 (0.70 – 1.10)	No
	LBW	RR = 0.67 (0.48 – 0.95)	Yes
da Silva et al. [9]	PB	RR = 0.54 (0.38 – 0.77)	Yes
	LBW	RR = 0.78 (0.5 – 1.21)	No
Schwendicke et al. [10]	PB	OR = 0.79 (0.57 – 1.10)	No
	LBW	OR = 0.69 (0.43 – 1.13)	No

Shah et al. [11]	PB and LBW	Two studies found significant differences in the incidence of LBW and four studies for PB.	Yes
Boutin et al. [12]	PB	RR = 0.89 (0.73 – 1.08)	No
	LBW	RR = 0.44 (0.31 – 0.65)	Yes
Kim et al. [13]	PB	RR = 0.66 (0.54 – 0.80)	Yes
	LBW	RR = 0.48 (0.30 – 0.78)	Yes
Rosa et al. [14]	PB	RR = 0.9 (0.68 – 1.19)	No
	LBW	RR = 0.92 (0.71 – 1.20)	No
Corbella et al. [15]	PB	OR = 1.01 (0.74 – 1.38)	No
	LBW	OR = 1.08 (0.86 – 1.36)	No
George et al. [16]	PB	OR = 0.65 (0.45 – 0.93)	Yes
	LBW	OR = 0.53 (0.31 – 0.92)	Yes
Fogacci et al. [17]	PB	RR = 0.92 (0.72 – 1.17)	No
	LBW	RR = 1.03 (0.76 – 1.40)	No
Chambrone et al. [18]	PB	RR = 0.88 (0.72 – 1.09)	No
	LBW	RR = 0.78 (0.53 – 1.17)	No
Polyzos et al. [19]	PB	OR = 0.93 (0.79 – 1.10)	No
	LBW	OR = 0.85 (0.70 – 1.04)	No
Uppal et al. [20]	PB	OR = 0.59 (0.39 – 0.88)	Yes
	LBW	OR = 0.72 (0.44 – 1.17)	No
Pimentel Lopes de Oliveira et al. [21]	PB and LBW	Reductions of PB ranged from 0.8% to 28.01%, while reduction of LBW ranged from 0.44% to 33%.	Yes
Polyzos et al. [22]	PB	OR = 0.55 (0.35 – 0.86)	Yes
	LBW	OR = 0.48 (0.23 – 1.00)	No
Xiong et al. [23]	PB and LBW	Three clinical trial studies indicate that oral prophylaxis and periodontal treatment may result in a 57% decrease in LBW cases and a 50% decrease in PB occurrences.	Yes
Scannapieco et al. [24]	PB and LBW	Periodontal treatment reduces PB and LBW	Yes

PB = preterm birth; LBW = low birth weight; OR = odds ratio; RR = risk/rate ratio.

Reference:

1. Alnasser, B.H.; Alkhaldi, N.K.; Alghamdi, W.K.; Alghamdi, F.T. The Potential Association Between Periodontal Diseases and Adverse Pregnancy Outcomes in Pregnant Women: A Systematic Review of Randomized Clinical Trials. *Cureus* **2023**, *15*, e33216, doi:10.7759/cureus.33216.
2. Orlandi, M.; Aguilera, E.; Marletta, D.; Petrie, A.; Suvan, J.; D'Aiuto, F. Impact of the Treatment of Periodontitis on Systemic Health and Quality of Life: A Systematic Review. *J. Clin. Periodontol.* **2022**, *49*, 314–327, doi:10.1111/jcpe.13554.
3. Merchant, A.T.; Gupta, R.D.; Akonde, M.; Reynolds, M.; Smith-Warner, S.; Liu, J.; Tarannum, F.; Beck, J.; Mattison, D. Association of Chlorhexidine Use and Scaling and Root Planing With Birth Outcomes in Pregnant Individuals With Periodontitis: A Systematic Review and Meta-Analysis. *JAMA Netw. Open* **2022**, *5*, e2247632, doi:10.1001/jamanetworkopen.2022.47632.
4. Le, Q.-A.; Eslick, G.D.; Coulton, K.M.; Akhter, R.; Lain, S.; Nassar, N.; Yaacoub, A.; Condous, G.; Leonardi, M.; Eberhard, J.; et al. Differential Impact of Periodontal Treatment Strategies during Pregnancy on Perinatal Outcomes: A Systematic Review and Meta-Analysis. *J. Evid.-Based Dent. Pract.* **2022**, *22*, 101666, doi:10.1016/j.jebdp.2021.101666.

5. Bi, W.G.; Emami, E.; Luo, Z.-C.; Santamaria, C.; Wei, S.Q. Effect of Periodontal Treatment in Pregnancy on Perinatal Outcomes: A Systematic Review and Meta-Analysis. *J. Matern.-Fetal Neonatal Med. Off. J. Eur. Assoc. Perinat. Med. Fed. Asia Ocean. Perinat. Soc. Int. Soc. Perinat. Obstet.* **2021**, *34*, 3259–3268, doi:10.1080/14767058.2019.1678142.
6. Le, Q.-A.; Eslick, G.D.; Coulton, K.M.; Akhter, R.; Condous, G.; Eberhard, J.; Nanan, R. Does Treatment of Gingivitis During Pregnancy Improve Pregnancy Outcomes? A Systematic Review and Meta-Analysis. *Oral Health Prev. Dent.* **2021**, *19*, 565–572, doi:10.3290/j.ohpd.b2183059.
7. Govindasamy, R.; Periyasamy, S.; Narayanan, M.; Balaji, V.R.; Dhanasekaran, M.; Karthikeyan, B. The Influence of Nonsurgical Periodontal Therapy on the Occurrence of Adverse Pregnancy Outcomes: A Systematic Review of the Current Evidence. *J. Indian Soc. Periodontol.* **2020**, *24*, 7–14, doi:10.4103/jisp.jisp_228_19.
8. Iheozor-Ejiofor, Z.; Middleton, P.; Esposito, M.; Glenny, A.-M. Treating Periodontal Disease for Preventing Adverse Birth Outcomes in Pregnant Women. *Cochrane Database Syst. Rev.* **2017**, *6*, CD005297, doi:10.1002/14651858.CD005297.pub3.
9. da Silva, H.E.C.; Stefani, C.M.; de Santos Melo, N.; de Almeida de Lima, A.; Rösing, C.K.; Porporatti, A.L.; Canto, G.D.L. Effect of Intra-Pregnancy Nonsurgical Periodontal Therapy on Inflammatory Biomarkers and Adverse Pregnancy Outcomes: A Systematic Review with Meta-Analysis. *Syst. Rev.* **2017**, *6*, doi:10.1186/s13643-017-0587-3.
10. Schwendicke, F.; Karimbux, N.; Allareddy, V.; Gluud, C. Periodontal Treatment for Preventing Adverse Pregnancy Outcomes: A Meta- and Trial Sequential Analysis. *PloS One* **2015**, *10*, e0129060, doi:10.1371/journal.pone.0129060.
11. Shah, M.; Muley, A.; Muley, P. Effect of Nonsurgical Periodontal Therapy during Gestation Period on Adverse Pregnancy Outcome: A Systematic Review. *J. Matern.-Fetal Neonatal Med. Off. J. Eur. Assoc. Perinat. Med. Fed. Asia Ocean. Perinat. Soc. Int. Soc. Perinat. Obstet.* **2013**, *26*, 1691–1695, doi:10.3109/14767058.2013.799662.
12. Boutin, A.; Demers, S.; Roberge, S.; Roy-Morency, A.; Chandad, F.; Bujold, E. Treatment of Periodontal Disease and Prevention of Preterm Birth: Systematic Review and Meta-Analysis. *Am. J. Perinatol.* **2013**, *30*, 537–544, doi:10.1055/s-0032-1329687.
13. Kim, A.J.; Lo, A.J.; Pullin, D.A.; Thornton-Johnson, D.S.; Karimbux, N.Y. Scaling and Root Planing Treatment for Periodontitis to Reduce Preterm Birth and Low Birth Weight: A Systematic Review and Meta-Analysis of Randomized Controlled Trials. *J. Periodontol.* **2012**, *83*, 1508–1519, doi:10.1902/jop.2012.110636.
14. Rosa, M.I. da; Pires, P.D.S.; Medeiros, L.R.; Edelweiss, M.I.; Martínez-Mesa, J. Periodontal Disease Treatment and Risk of Preterm Birth: A Systematic Review and Meta-Analysis. *Cad. Saúde Pública* **2012**, *28*, 1823–1833, doi:10.1590/S0102-311X2012001000002.
15. Corbella, S.; Del Fabbro, M.; Taschieri, S.; Francetti, L. Periodontal Disease and Adverse Pregnancy Outcomes: A Systematic Review. *Ital. Oral Surg.* **2012**, *11*, 132–146, doi:10.1016/j.ios.2011.04.002.
16. George, A.; Shamim, S.; Johnson, M.; Ajwani, S.; Bhole, S.; Blinkhorn, A.; Ellis, S.; Andrews, K. Periodontal Treatment during Pregnancy and Birth Outcomes: A Meta-Analysis of Randomised Trials. *Int. J. Evid. Based Healthc.* **2011**, *9*, 122–147, doi:10.1111/j.1744-1609.2011.00210.x.
17. Fogacci, M.F.; Vettore, M.V.; Thomé Leão, A.T. The Effect of Periodontal Therapy on Preterm Low Birth Weight: A Meta-Analysis. *Obstet. Gynecol.* **2011**, *117*, 153–165, doi:10.1097/AOG.0b013e3181fdebc0.
18. Chambrone, L.; Pannuti, C.M.; Guglielmetti, M.R.; Chambrone, L.A. Evidence Grade Associating Periodontitis with Preterm Birth and/or Low Birth Weight: II. A Systematic Review

of Randomized Trials Evaluating the Effects of Periodontal Treatment. *J. Clin. Periodontol.* **2011**, *38*, 902–914, doi:10.1111/j.1600-051X.2011.01761.x.
19. Polyzos, N.P.; Polyzos, I.P.; Zavos, A.; Valachis, A.; Mauri, D.; Papanikolaou, E.G.; Tzioras, S.; Weber, D.; Messinis, I.E. Obstetric Outcomes after Treatment of Periodontal Disease during Pregnancy: Systematic Review and Meta-Analysis. *BMJ* **2010**, *341*, c7017, doi:10.1136/bmj.c7017.
20. Uppal, A.; Uppal, S.; Pinto, A.; Dutta, M.; Shrivatsa, S.; Dandolu, V.; Mupparapu, M. The Effectiveness of Periodontal Disease Treatment during Pregnancy in Reducing the Risk of Experiencing Preterm Birth and Low Birth Weight: A Meta-Analysis. *J. Am. Dent. Assoc. 1939* **2010**, *141*, 1423–1434, doi:10.14219/jada.archive.2010.0104.
21. Pimentel Lopes De Oliveira, G.J.; Amaral Fontanari, L.; Chaves De Souza, J.A.; Ribeiro Costa, M.; Cirelli, J.A. Effect of Periodontal Treatment on the Incidence of Preterm Delivery: A Systematic Review. *Minerva Stomatol.* **2010**, *59*, 543–550.
22. Polyzos, N.P.; Polyzos, I.P.; Mauri, D.; Tzioras, S.; Tsappi, M.; Cortinovis, I.; Casazza, G. Effect of Periodontal Disease Treatment during Pregnancy on Preterm Birth Incidence: A Metaanalysis of Randomized Trials. *Am. J. Obstet. Gynecol.* **2009**, *200*, 225–232, doi:10.1016/j.ajog.2008.09.020.
23. Xiong, X.; Buekens, P.; Fraser, W.D.; Beck, J.; Offenbacher, S. Periodontal Disease and Adverse Pregnancy Outcomes: A Systematic Review. *BJOG Int. J. Obstet. Gynaecol.* **2006**, *113*, 135–143, doi:10.1111/j.1471-0528.2005.00827.x.
24. Scannapieco, F.A.; Bush, R.B.; Paju, S. Periodontal Disease as a Risk Factor for Adverse Pregnancy Outcomes. A Systematic Review. *Ann. Periodontol.* **2003**, *8*, 70–78, doi:10.1902/annals.2003.8.1.70.
25. López, N.J.; Smith, P.C.; Gutierrez, J. Periodontal Therapy May Reduce the Risk of Preterm Low Birth Weight in Women with Periodontal Disease: A Randomized Controlled Trial. *J. Periodontol.* **2002**, *73*, 911–924, doi:10.1902/jop.2002.73.8.911.
26. Tarannum, F.; Faizuddin, M. Effect of Periodontal Therapy on Pregnancy Outcome in Women Affected by Periodontitis. *J. Periodontol.* **2007**, *78*, 2095–2103, doi:10.1902/jop.2007.060388.
27. Michalowicz, B.S.; Hodges, J.S.; DiAngelis, A.J.; Lupo, V.R.; Novak, M.J.; Ferguson, J.E.; Buchanan, W.; Bofill, J.; Papapanou, P.N.; Mitchell, D.A.; et al. Treatment of Periodontal Disease and the Risk of Preterm Birth. *N. Engl. J. Med.* **2006**, *355*, 1885–1894, doi:10.1056/NEJMoa062249.
28. Sadatmansouri, S.; Sedighpoor, N.; Aghaloo, M. Effects of Periodontal Treatment Phase I on Birth Term and Birth Weight. *J. Indian Soc. Pedod. Prev. Dent.* **2006**, *24*, 23–26, doi:10.4103/0970-4388.22831.
29. Newnham, J.P.; Newnham, I.A.; Ball, C.M.; Wright, M.; Pennell, C.E.; Swain, J.; Doherty, D.A. Treatment of Periodontal Disease during Pregnancy: A Randomized Controlled Trial. *Obstet. Gynecol.* **2009**, *114*, 1239–1248, doi:10.1097/AOG.0b013e3181c15b40.
30. Offenbacher, S.; Lin, D.; Strauss, R.; McKaig, R.; Irving, J.; Barros, S.P.; Moss, K.; Barrow, D.A.; Hefti, A.; Beck, J.D. Effects of Periodontal Therapy during Pregnancy on Periodontal Status, Biologic Parameters, and Pregnancy Outcomes: A Pilot Study. *J. Periodontol.* **2006**, *77*, 2011–2024, doi:10.1902/jop.2006.060047.
31. Offenbacher, S.; Beck, J.D.; Jared, H.L.; Mauriello, S.M.; Mendoza, L.C.; Couper, D.J.; Stewart, D.D.; Murtha, A.P.; Cochran, D.L.; Dudley, D.J.; et al. Effects of Periodontal Therapy on Rate of Preterm Delivery: A Randomized Controlled Trial. *Obstet. Gynecol.* **2009**, *114*, 551–559, doi:10.1097/AOG.0b013e3181b1341f.
32. Jeffcoat, M.K.; Hauth, J.C.; Geurs, N.C.; Reddy, M.S.; Cliver, S.P.; Hodgkins, P.M.; Goldenberg, R.L. Periodontal Disease and Preterm Birth: Results of a Pilot Intervention Study. *J. Periodontol.* **2003**, *74*, 1214–1218, doi:10.1902/jop.2003.74.8.1214.

33. Radnai, M.; Pál, A.; Novák, T.; Urbán, E.; Eller, J.; Gorzó, I. Benefits of Periodontal Therapy When Preterm Birth Threatens. *J. Dent. Res.* **2009**, *88*, 280–284, doi:10.1177/0022034508330229.
34. López, N.J.; Da Silva, I.; Ipinza, J.; Gutiérrez, J. Periodontal Therapy Reduces the Rate of Preterm Low Birth Weight in Women with Pregnancy-Associated Gingivitis. *J. Periodontol.* **2005**, *76*, 2144–2153, doi:10.1902/jop.2005.76.11-S.2144.
35. Macones, G.A.; Parry, S.; Nelson, D.B.; Strauss, J.F.; Ludmir, J.; Cohen, A.W.; Stamilio, D.M.; Appleby, D.; Clothier, B.; Sammel, M.D.; et al. Treatment of Localized Periodontal Disease in Pregnancy Does Not Reduce the Occurrence of Preterm Birth: Results from the Periodontal Infections and Prematurity Study (PIPS). *Am. J. Obstet. Gynecol.* **2010**, *202*, 147.e1-8, doi:10.1016/j.ajog.2009.10.892.
36. Oliveira, A.M.S.D.; de Oliveira, P.A.D.; Cota, L.O.M.; Magalhães, C.S.; Moreira, A.N.; Costa, F.O. Periodontal Therapy and Risk for Adverse Pregnancy Outcomes. *Clin. Oral Investig.* **2011**, *15*, 609–615, doi:10.1007/s00784-010-0424-8.
37. Pirie, M.; Linden, G.; Irwin, C. Intrapregnancy Non-Surgical Periodontal Treatment and Pregnancy Outcome: A Randomized Controlled Trial. *J. Periodontol.* **2013**, *84*, 1391–1400, doi:10.1902/jop.2012.120572.
38. Weidlich, P.; Moreira, C.H.C.; Fiorini, T.; Musskopf, M.L.; da Rocha, J.M.; Oppermann, M.L.R.; Aass, A.M.; Gjermo, P.; Susin, C.; Rösing, C.K.; et al. Effect of Nonsurgical Periodontal Therapy and Strict Plaque Control on Preterm/Low Birth Weight: A Randomized Controlled Clinical Trial. *Clin. Oral Investig.* **2013**, *17*, 37–44, doi:10.1007/s00784-012-0679-3.
39. Jeffcoat, M.; Parry, S.; Sammel, M.; Clothier, B.; Catlin, A.; Macones, G. Periodontal Infection and Preterm Birth: Successful Periodontal Therapy Reduces the Risk of Preterm Birth. *BJOG Int. J. Obstet. Gynaecol.* **2011**, *118*, 250–256, doi:10.1111/j.1471-0528.2010.02713.x.
40. Gazolla, C.M.; Ribeiro, A.; Moysés, M.R.; Oliveira, L.A.M.; Pereira, L.J.; Sallum, A.W. Evaluation of the Incidence of Preterm Low Birth Weight in Patients Undergoing Periodontal Therapy. *J. Periodontol.* **2007**, *78*, 842–848, doi:10.1902/jop.2007.060295.
41. Khairnar, M.S.; Pawar, B.R.; Marawar, P.P.; Khairnar, D.M. Estimation of Changes in C-Reactive Protein Level and Pregnancy Outcome after Nonsurgical Supportive Periodontal Therapy in Women Affected with Periodontitis in a Rural Set up of India. *Contemp. Clin. Dent.* **2015**, *6*, S5–S11, doi:10.4103/0976-237X.152930.
42. Herrera, J.A.; Vélez-Medina, S.; Molano, R.; Medina, V.; Botero, J.E.; Parra, B.; Contreras, A. Periodontal Intervention Effects on Pregnancy Outcomes in Women with Preeclampsia. *Colomb. Médica* **2009**, *40*, 177–184.
43. Sant'Ana, A.C.P.; Campos, M.R. de; Passanezi, S.C.; Rezende, M.L.R. de; Greghi, S.L.A.; Passanezi, E. Periodontal Treatment during Pregnancy Decreases the Rate of Adverse Pregnancy Outcome: A Controlled Clinical Trial. *J. Appl. Oral Sci. Rev. FOB* **2011**, *19*, 130–136, doi:10.1590/s1678-77572011000200009.
44. Caneiro-Queija, L.; López-Carral, J.; Martin-Lancharro, P.; Limeres-Posse, J.; Diz-Dios, P.; Blanco-Carrion, J. Non-Surgical Treatment of Periodontal Disease in a Pregnant Caucasian Women Population: Adverse Pregnancy Outcomes of a Randomized Clinical Trial. *Int. J. Environ. Res. Public. Health* **2019**, *16*, 3638, doi:10.3390/ijerph16193638.
45. Reddy, B.V.R.; Tanneeru, S.; Chava, V.K. The Effect of Phase-I Periodontal Therapy on Pregnancy Outcome in Chronic Periodontitis Patients. *J. Obstet. Gynaecol. J. Inst. Obstet. Gynaecol.* **2014**, *34*, 29–32, doi:10.3109/01443615.2013.829029.
46. Mitchell-Lewis, D.; Engebretson, S.P.; Chen, J.; Lamster, I.B.; Papapanou, P.N. Periodontal Infections and Pre-Term Birth: Early Findings from a Cohort of Young Minority Women in New York. *Eur. J. Oral Sci.* **2001**, *109*, 34–39, doi:10.1034/j.1600-0722.2001.00966.x.

47. Farrell, S. Investigation of the Effect of Treatment of Maternal Chronic Periodontitis on Delivery and Low Birth Weight. *Res. Find. Regist. ReFeR* **2003**.
48. Novák, T.; Radnai, M.; Gorzó, I.; Urbán, E.; Orvos, H.; Eller, J.; Pál, A. Prevention of Preterm Delivery with Periodontal Treatment. *Fetal Diagn. Ther.* **2009**, *25*, 230–233, doi:10.1159/000221378.
49. Penova-Veselinovic, B.; Keelan, J.A.; Wang, C.A.; Newnham, J.P.; Pennell, C.E. Changes in Inflammatory Mediators in Gingival Crevicular Fluid Following Periodontal Disease Treatment in Pregnancy: Relationship to Adverse Pregnancy Outcome. *J. Reprod. Immunol.* **2015**, *112*, 1–10, doi:10.1016/j.jri.2015.05.002.
50. Novák, T.; Radnai, M.; Kozinszky, Z.; Práger, N.; Hodoniczki, L.; Gorzó, I.; Németh, G. [Effect of the treatment of periodontal disease on the outcome of pregnancy]. *Orv. Hetil.* **2018**, *159*, 978–984, doi:10.1556/650.2018.31103.
